# 309. Genetic Diversity and Conservation of Envelope Gene of SARS-CoV-2 and its Variants B.1.1.7, B.1.351, P.1, B.1.617.2 and B.1.1.529 with comparison with other human coronaviruses

**DOI:** 10.1093/ofid/ofac492.387

**Published:** 2022-12-15

**Authors:** Benjamin Liu

**Affiliations:** Children's National Hospital, George Washington University, Washington, District of Columbia

## Abstract

**Background:**

SARS-CoV-2 E gene PCRs have been widely used as the first-line assay with a higher sensitivity than those targeting N or RdRp gene. Given the currently available primers and probes were designed at the onset of the pandemic, it is unknown whether the SARS-CoV-2 VOCs have accumulated significant mutations that may affect E gene PCRs. In this study we aim to perform a comprehensive genetic analysis of SARS-CoV-2 E gene sequences to evaluate the impact of the emerging VOCs on E gene PCR performance.

**Methods:**

600 whole-genome sequences of 7 species of human coronaviruses (HCoVs) were retrieved from GenBank and GISAID, including Sarbecoviruses (SARS-CoV-2 variants B1.1.7, B1.351, P.1, B.1.617.2 and B.1.1.529, and SARS-CoV), Embecovirus (OC43, HKU1), Merbecovirus (MERS) and Alphacoronaviruses (229E, NL63). The E gene sequences were retrieved from full-length genomes of corresponding viruses and aligned by ClustalW multiple alignment. Phylogenetic, conservation and mutation analyses analysis of the enrolled sequences was performed.

**Results:**

E gene-based phylogenetic analysis yielded HCoVs typing results consistent with whole genome typing, suggesting E gene is a reliable locus for phylogenetic analysis and typing of HCoVs. Four pan-Sarbecovirus conserved E gene regions were identified with 100% conserved nucleotide similarity among SARS-CoV-2 and its VOCs, as well as SARS-CoV. These regions have appropriate G/C content which may be suitable for primer/probe design for E gene-based pan-Sarbecovirus screening assay. No significant E mutations were found in 137 retrieved SARS-CoV-2 and its VOCs. Interestingly, two novel variations, C26299U and T26354A, were identified in two of our SARS-CoV-2 strains. The latter variation occurred at the 3’ end of the target region of the widely used Charité/Berlin (WHO) probe. This variant may lead to a potential failure of the first-line E gene PCR.

**Conclusion:**

Our data shed light on the genetic diversity and conservation of E gene of SARS-CoV-2 and may be beneficial for future primer/probe design for novel first-line assay or SARS-CoV-2-specific E gene PCR. SARS-CoV-2 VOCs have not accumulated significant mutations in E gene so far. The impact of novel E gene variations C26299U and T26354A on molecular diagnostic testing warrants further investigation.

**Disclosures:**

**All Authors**: No reported disclosures.

